# Preoperative Risk Stratification of Cerebral Infarction in Aortic Arch Surgery Using Quantitative Atheroma Assessment with Computational Fluid Dynamics

**DOI:** 10.5761/atcs.oa.26-00102

**Published:** 2026-08-14

**Authors:** Hiroki Ikeuchi, Hiroki Kohno, Kaoru Matsuura, Michiko Watanabe, Tomohiko Inui, Yasunori Yakita, Goro Matsumiya

**Affiliations:** Department of Cardiovascular Surgery, Chiba University Graduate School of Medicine, Chiba, Chiba, Japan

**Keywords:** aortic arch surgery, computed tomography, atherosclerotic change, postoperative cerebral infarction, computational fluid dynamics

## Abstract

**Purpose:**

We developed a quantitative method to evaluate aortic atheroma and investigated its association with postoperative cerebral infarction (CI) after aortic arch surgery. In addition, computational fluid dynamics (CFD) analysis was performed to explore the underlying hemodynamic mechanisms.

**Methods:**

We retrospectively analyzed 72 patients who underwent aortic arch surgery. Atheroma scores were calculated from preoperative computed tomography, and a qualitative assessment was also performed. Multivariate logistic regression identified risk factors for CI. CFD analysis was conducted in selected patients to provide mechanistic insights.

**Results:**

CI occurred in 8 patients (11.1%). Atheroma scores were significantly higher in patients with CI (P = .02), whereas qualitative assessment did not predict CI. Receiver operating characteristic analysis showed an area under the curve of 0.79 for the ascending aorta, with a cutoff of 352.3. Multivariate analysis including age demonstrated that hemoglobin A1c and an ascending atheroma score ≥352.3 were independent predictors of CI. CFD demonstrated high wall shear stress in the ascending aorta, providing mechanistic insight into embolic events.

**Conclusions:**

Quantitative assessment of ascending aortic atheroma provides superior risk stratification compared with qualitative evaluation. CFD findings offer mechanistic insight into embolic risk, supporting the clinical relevance of atheroma burden in aortic arch surgery.

## Introduction

The number of patients undergoing surgery for thoracic aortic aneurysms is increasing with the aging population.^1)^ Although endovascular and hybrid approaches have advanced, open surgery under cardiopulmonary bypass (CPB) remains the gold standard for aortic arch aneurysms.

Neurologic complications remain one of the most serious adverse events in aortic arch surgery and are associated with increased mortality and morbidity.^2–4)^ Despite advances in cerebral protection strategies, including antegrade cerebral perfusion and subclavian artery (SCA) cannulation, the incidence of postoperative cerebral infarction (CI) remains non-negligible.

Atherosclerotic changes in the aortic wall are frequently observed in elderly patients and have been recognized as a major source of embolism during arch surgery.^5)^ While enhanced computed tomography (CT) allows preoperative evaluation of atherosclerosis, its assessment remains largely subjective, and no standardized quantitative method has been established to objectively stratify stroke risk in open aortic arch surgery.

In addition to plaque burden, intraoperative hemodynamic factors, particularly high-velocity perfusion jets during CPB, may contribute to embolic events; however, the underlying mechanisms are not fully understood. Computational fluid dynamics (CFD) enables detailed evaluation of hemodynamic parameters, including flow patterns and wall shear stress (WSS).^6,7)^ Elevated WSS has been associated with plaque destabilization and rupture.^8)^

In this study, we developed a quantitative method for assessing aortic atheroma and investigated its association with postoperative CI. Furthermore, we used CFD analysis to explore the hemodynamic mechanisms underlying embolic complications.

## Materials and Methods

### Study design

This was a retrospective cohort study conducted at a single institution between January 2010 and December 2019. During this period, 111 patients underwent aortic arch surgery. Patients who underwent emergency surgery, surgery for aortic dissection, an infectious aneurysm, or an inflammatory aneurysm were excluded. Ultimately, 72 patients were included in this study. All patients underwent preoperative brain magnetic resonance imaging (MRI).

The study was approved by the institutional review board of our hospital (Protocol Nos. 1250 and 3507; approval dates: June 26, 2017, and January 23, 2020). The requirement for individual informed consent was waived.

### Surgical techniques

All 72 patients underwent total arch replacement using a 4-branched graft via median sternotomy. To reduce the risk of cerebral embolism, right SCA cannulation and antegrade cerebral perfusion were routinely employed.^4)^ No ascending aortic clamping was performed before circulatory arrest.

After cooling to a rectal temperature of 26°C, circulatory arrest was initiated, and the aneurysm was opened. Selective cerebral perfusion was established by clamping the innominate artery and inserting balloon-tipped cannulas into the left common carotid artery and the left SCA. Cerebral perfusion was maintained at 10–12 mL/kg/min.

Following the distal anastomosis, lower body perfusion was resumed via a branch graft, and rewarming was initiated. The proximal anastomosis was then performed, followed by coronary reperfusion and reconstruction of the arch vessels. Frozen elephant trunk was used in 10 patients (13.9%).

### Definition of neurologic outcomes

Neurologic dysfunction was defined as a permanent neurologic deficit, including symptoms such as paralysis or dysarthria, persisting at discharge. Postoperative CI was diagnosed by a neurologist based on imaging findings (MRI or CT) in patients presenting with new neurologic symptoms.

### Analysis of atherosclerotic changes

**Fig. 1:**
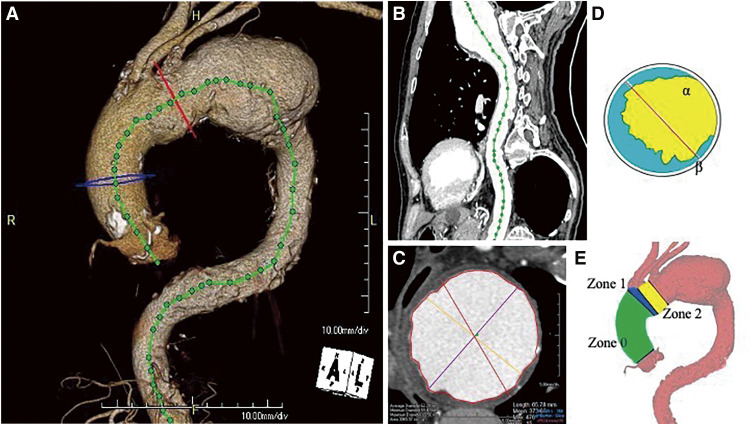
Quantitative assessment of aortic atheroma using CT. (**A**, **B**) Three-dimensional reconstruction of CT images and generation of the aortic centerline (green line) using curved multiplanar reconstruction. (**C**) Cross-sectional analysis perpendicular to the centerline. The luminal area (α) was automatically calculated for each slice. (**D**) Schematic illustration of atheroma area calculation. The atheroma area was defined as the difference between the theoretical circular area based on the maximum vessel diameter (β) and the actual luminal area (α). (**E**) Segmentation of the aorta into 3 regions (zones 0–2) for regional analysis of atheroma burden. CT, computed tomography

Atherosclerotic changes were evaluated using multidetector-row CT. Imaging data were reconstructed into three-dimensional (3D) models (**Fig. 1A**) using a dedicated workstation (AquariusNET; TeraRecon, Foster City, CA, USA). A centerline was generated from the aortic root to the descending aorta (**Fig. 1B**), and cross-sectional images perpendicular to the centerline were automatically obtained (**Fig. 1C**).

The cross-sectional lumen area (α) was calculated for each slice, and a theoretical circular area based on the maximum vessel diameter (β) was defined. The atheroma area was calculated as the difference between β and α (**Fig. 1D**).

$$Atheroma\;\;area={\left(\frac{\text{β}}{2}\right)}^{2}\pi -\text{α}$$

The atheroma area was measured at 10-mm intervals from the sinotubular junction to the orifice of the left SCA. The atheroma score was defined as the sum of the atheroma areas. Because the atheroma score represents the total plaque burden, it is influenced by the length of each aortic segment; therefore, comparisons between segments should be interpreted with caution. The aorta was divided into 3 segments (zones 0–2), and segmental atheroma scores were calculated (**Fig. 1E**). As a sensitivity analysis, a length-normalized atheroma score was calculated by dividing the segmental atheroma score by the number of measured cross-sectional slices in each segment. Because measurements were performed at 10-mm intervals, this value represented the average atheroma area per 10-mm segment.

Qualitative assessment of atherosclerosis was performed by determining the presence of a shaggy aorta on preoperative enhanced CT. In this study, a shaggy aorta was defined as diffuse, irregular atheromatous change with protruding plaque and/or mural thrombus in the thoracic aorta. The anatomical distribution of the shaggy aorta was assessed across the ascending aorta, aortic arch, and descending thoracic aorta. This qualitative assessment was performed for the thoracic aorta as a whole and was not limited to a specific aortic segment.

All measurements were performed by a single experienced surgeon blinded to the clinical outcomes.

### CFD analysis

CFD analysis was performed in 3 of the 8 patients with postoperative CI for whom electrocardiography-gated CT provided sufficient image quality for accurate reconstruction of the aortic root and ascending aorta. The purpose of the CFD analysis was to explore hemodynamic characteristics and provide mechanistic insight into embolic events. These cases were selected for illustrative and hypothesis-generating analysis and were not intended to establish a predictive CFD model.

### Geometry and mesh

Data from enhanced CT imaging were transferred to another 3D workstation (Ziostation 2; Ziosoft, Tokyo, Japan) and converted to 3D data (STL format). With reference to a previous CFD analysis,^7)^ all inlet and outlet boundaries were extended up to 50 times the diameter of each vessel in order to get a stable flow using modeling software (The Vascular Modeling Tool Kit; Orobix, Bergamo, Italy). Tetrahedral meshes were created using ANSYS-ICEM CFD 14.5 (ANSYS Japan, Tokyo, Japan). Since the typical Reynolds number, which expresses the motion of fluid, is approximately 4000 in the aorta,^9)^ it was necessary to create fine meshes and perform a turbulence analysis. According to a previous report, meshes containing more than 2000000 cells with 5 boundary layers were generated.^10)^ The distance of the first layer to the vessel wall was fixed at 0.02 mm.

### Boundary conditions and turbulent analysis

The perfusion flow from the right SCA was set to 5.0 L/min as a steady flow in the right SCA perfusion model. Similarly, perfusion flow through the right and left SCA was set to 2.5 L/min in the bilateral SCA perfusion model. All outlet boundary conditions were fixed at the same barometric pressure. The bilateral SCA perfusion model was constructed as a hypothetical simulation by modifying the inlet boundary conditions; no patient in this cohort actually underwent bilateral SCA perfusion. In the right SCA perfusion model, a steady inflow of 5.0 L/min was assigned to the right SCA. In the bilateral SCA perfusion model, the same total flow of 5.0 L/min was divided equally between the right and left SCAs, with 2.5 L/min assigned to each side. The cannula itself was not explicitly modeled; instead, inflow boundaries were placed at the proximal SCA, and flow was directed along the vessel axis. These flow rates were used as standardized model conditions and were not adjusted according to patient-specific body surface area.

CFD simulations were carried out using ANSYS-CFX 14.5 (ANSYS Japan, Tokyo, Japan). Blood was modeled as an incompressible Newtonian fluid. The density and viscosity of the blood were 1060 kg/m^3^ and 0.0035 Pa/s, respectively. The blood flow was modeled using the Navier-Stokes equation. The RNG k-epsilon model was selected to analyze turbulent flow simulation. The time step was set to 0.001 s, and the convergence criterion was set to 10−5. To obtain reliable results, all analyses were performed for 2 s, and the last half of the data were extracted as the result.

### Statistical analysis

Continuous variables were expressed as the mean ± standard deviation. Normality was assessed using the Shapiro–Wilk test. Continuous variables were compared using Student’s t-test or the Mann–Whitney U test, as appropriate. Categorical variables were analyzed using the chi-square test or Fisher’s exact test.

Multivariate logistic regression analysis was performed to identify independent risk factors for postoperative CI. Receiver operating characteristic curves were generated, and the optimal cutoff values were determined using the Youden index. A P value <.05 was considered statistically significant. Variables with P <.05 in the univariate analysis were entered into the multivariate model, and age was additionally included because of its known clinical relevance to CI.

All statistical analyses were performed using JMP Pro 14.2 (SAS Institute, Cary, NC, USA).

## Results

### Overall outcomes

Postoperative neurologic dysfunction occurred in 8 patients (11.1%), all of whom were diagnosed with CI. Among these, 7 patients had multiple bilateral cerebral embolic lesions. Patients were divided into 2 groups according to the presence or absence of postoperative CI.

### Patient characteristics

Patient characteristics are summarized in **Table 1**. The mean age was 73.0 ± 5.6 years and was significantly higher in patients with CI (76.5 ± 2.8 vs 72.5 ± 5.7 years, P = .02). Saccular aneurysms were present in 49 patients (68.1%). Hemoglobin A1c levels were significantly higher in patients with CI (P <.01). Leukoaraiosis on MRI tended to be more severe in the CI group, although the difference was not statistically significant. Other MRI findings were comparable between the 2 groups.

**Table 1:** Patient characteristics

Variables	Overall (n = 72)	Postoperative CI group (n = 8)	Non-postoperative CI group (n = 64)	P value
Age, y	73.0 ± 5.6	76.5 ± 2.8	72.5 ± 5.7	0.02
Male	56 (77.8)	7 (87.5)	49 (76.6)	0.48
Hypertension	60 (83.3)	6 (75.0)	54 (84.4)	0.5
Dyslipidemia	46 (63.9)	5 (62.5)	41 (64.1)	0.93
Total cholesterol, mg/dL	180.7 ± 31.2	182.5 ± 11.1	180.5 ± 3.9	0.87
Diabetes mellitus	11 (15.3)	3 (37.5)	8 (12.5)	0.1
Hemoglobin A1c, %	5.8 ± 0.7	6.5 ± 0.2	5.7 ± 0.1	<0.01
Chronic obstructive pulmonary disease	61 (84.7)	7 (87.5)	54 (84.4)	1
Chronic kidney disease	37 (51.4)	4 (50.0)	33 (51.6)	1
Peripheral artery disease	11 (15.3)	1 (12.5)	10 (15.6)	1
Carotid artery stenosis	11 (15.3)	1 (12.5)	10 (15.6)	1
Intracranial artery stenosis	3 (4.2)	0 (0.0)	3 (4.2)	1
Leukoaraiosis	52 (72.2)	8 (100)	44 (68.7)	0.09
Old CI	34 (47.2)	3 (37.5)	31 (48.4)	0.71
Saccular aneurysm	49 (68.1)	6 (75.0)	43 (67.2)	1
Saccular aneurysm of lesser curvature	7 (9.7)	1 (12.5)	6 (9.4)	0.58
Saccular aneurysm of greater curvature	11 (15.3)	2 (25.0)	9 (14.1)	0.6

CI, cerebral infarction

### Operative details and early outcomes

Operative details and early outcomes are shown in **Table 2**. CPB time, myocardial ischemic time, and the rate of concomitant procedures were similar between the 2 groups.

**Table 2:** Operative data and early outcomes

Variables	Overall (n = 72)	Postoperative CI group (n = 8)	Non-postoperative CI group (n = 64)	P value
Operation time, min	489.2 ± 101.0	548.1 ± 129.5	481.8 ± 95.6	0.09
CPB time, min	223.9 ± 47.2	251.1 ± 67.9	220.5 ± 43.5	0.18
Myocardial ischemic time, min	99.7 ± 29.0	91.9 ± 31.2	100.6 ± 28.9	0.64
Lower body circulatory arrest time, min	60.2 ± 19.5	61.8 ± 27.4	60.0 ± 18.6	0.24
Concomitant surgery				
Coronary artery bypass grafting	24 (33.3)	2 (25.0)	22 (34.4)	0.71
Aortic valve replacement	8 (11.1)	1 (12.5)	7 (10.9)	1
Frozen elephant trunk	10 (13.9)	2 (25.0)	8 (12.5)	0.31
Operative mortality	2 (2.8)	0 (0)	2 (3.1)	1
Postoperative atrial fibrillation	35 (48.6)	4 (50.0)	31 (48.4)	1
Deep sternal wound infection	2 (2.8)	0 (0)	2 (3.1)	1
Acute kidney injury	8 (11.1)	0 (0)	8 (12.5)	0.58
Blue toe syndrome	3 (4.2)	0 (0)	3 (4.7)	1
Paraplegia/paraparesis	3 (4.2)	0 (0)	3 (4.7)	1
Pneumonia	15 (20.8)	7 (87.5)	8 (12.5)	<0.01

CI, cerebral infarction

The operative mortality rate was 2.8% (2/72 patients), including 1 case of multisystem organ failure and 1 case of rupture of a descending aortic aneurysm. The occurrence of CI was not associated with operative mortality. Paraparesis and blue toe syndrome occurred in 3 patients (4.2%), with no association with CI.

### Atheroma score

The results of the atherosclerotic assessment are summarized in **Table 3**. The presence of a shaggy aorta was not associated with postoperative CI. The atheroma score was significantly higher in patients with CI (1351.5 ± 1187.5 vs 592.9 ± 669.8, P = .02). Segmental analysis demonstrated that atheroma scores in zones 0 and 2 were significantly higher in patients with CI (zone 0, P <.01; zone 2, P = .04).

**Table 3:** Analysis of the atheroma score

Variables	Overall (n = 72)	Postoperative CI group (n = 8)	Non-postoperative CI group (n = 64)	P value
Shaggy aorta	13 (18.1)	2 (25.0)	11 (17.2)	0.63
The atheroma score	677.2 ± 771.2	1351.5 ± 1187.5	592.9 ± 669.8	0.02
The atheroma score in Zone 0	279.3 ± 386.9	582.6 ± 477.7	241.4 ± 360.9	<0.01
The atheroma score in Zone 1	68.9 ± 136.1	223.8 ± 282.6	49.5 ± 92.6	0.12
The atheroma score in Zone 2	154.4 ± 275.4	287.9 ± 269.4	137.8 ± 273.6	0.04

CI, cerebral infarction

**Fig. 2:**
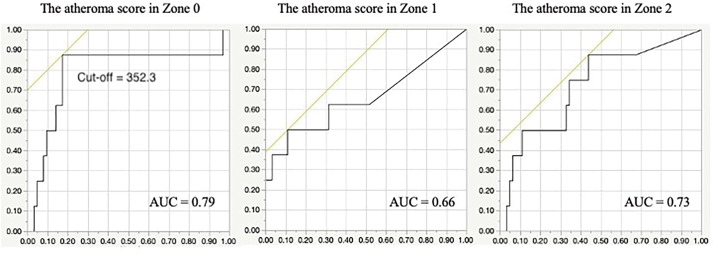
ROC curves of atheroma scores for predicting postoperative CI. ROC curves for atheroma scores in zone 0 (**A**), zone 1 (**B**), and zone 2 (**C**). The area under the curve was 0.79, 0.66, and 0.73, respectively. CI, cerebral infarction; ROC, receiver operating characteristic

Receiver operating characteristic analysis demonstrated that the area under the curve was 0.79 for zone 0, 0.66 for zone 1, and 0.73 for zone 2 (**Fig. 2**). The optimal cutoff values were 352.3 in zone 0 (sensitivity 87.5%, specificity 82.8%), 126.7 in zone 1, and 71.8 in zone 2. In a sensitivity analysis using the length-normalized atheroma score, the normalized score in zone 0 remained significantly higher in patients with CI than in those without CI (56.8 ± 42.5 vs 26.9 ± 41.2, P = .007). The area under the curve for the length-normalized zone 0 score was 0.785 (**Supplementary Table**).

### Risk analysis of postoperative CI

Univariate and multivariate analyses are presented in **Table 4**. Univariate analysis identified higher hemoglobin A1c levels and elevated atheroma scores as significant risk factors for CI.

**Table 4:** Univariate and multivariate analyses

Variables	Univariate analysis	P value	Multivariate analysis	P value
	OR (95% CI)		OR (95% CI)	
Age, y	1.14 (0.99–1.31)	0.06	1.14 (0.92–1.41)	0.24
BMI, kg/m2	1.18 (0.95–1.46)	0.13		
Male	2.14 (0.24 –18.8)	0.46		
Hypertension	0.56 (0.10–3.15)	0.52		
Dyslipidemia	0.93 (0.20–4.27)	0.93		
Total cholesterol, mg/dL	1.002 (0.98–1.03)	0.86		
Diabetes mellitus	4.20 (0.84–21.0)	0.1		
Hemoglobin A1c, %	3.45 (1.31–9.11)	0.01	17.11 (1.34–219.15)	0.029
Chronic obstructive pulmonary disease	1.30 (0.14–11.71)	0.81		
Chronic kidney disease	0.94 ( 0.22–4.09)	0.93		
Peripheral artery disease	0.77 (0.09–6.97)	0.81		
Ejection fraction	1.04 (0.93–1.16)	0.47		
Carotid artery stenosis	0.77 (0.09–6.97)	0.81		
Intracranial artery stenosis	–	0.4		
Old CI	0.64 (0.14–2.90)	0.56		
Saccular aneurysm	1.47 (0.27–7.89)	0.65		
Saccular aneurysm of lesser curvature	1.38 (0.14–13.20)	0.79		
Saccular aneurysm of greater curvature	2.04 (0.35–11.71)	0.45		
Concomitant coronary artery bypass grafting	0.64 (0.12–3.42)	0.59		
The atheroma score in Zone 0 ≥352.3	14.5 (2.57–81.29)	<0.01	84.04 (1.30–5440.76)	0.037
The atheroma score in Zone 1 ≥126.7	8.14 (1.65–40.04)	0.01	6.66 (0.49–90.10)	0.154
The atheroma score in Zone 2 ≥71.8	9.59 (1.11–82.61)	0.01	2.47 (0.17–36.57)	0.511

CI, cerebral infarction

Multivariate analysis including age demonstrated that hemoglobin A1c (OR 17.11, 95% CI 1.34–219.15, P = .029) and an atheroma score ≥352.3 in zone 0 (OR 84.04, 95% CI 1.30–5440.76, P = .037) were independent predictors of CI, whereas age was not independently associated with CI (OR 1.14, 95% CI 0.92–1.41, P = .24). No significant correlation was observed between hemoglobin A1c and the total atheroma score (Spearman ρ = −0.012, P = .92) or the zone 0 atheroma score (Spearman ρ = −0.019, P = .87).

### CFD analysis

CFD analysis demonstrated that blood flow from the right SCA was directed along the greater curvature of the ascending aorta toward the aortic root (**Supplementary Movie**). High WSS was predominantly observed in the ascending aorta.

**Fig. 3:**
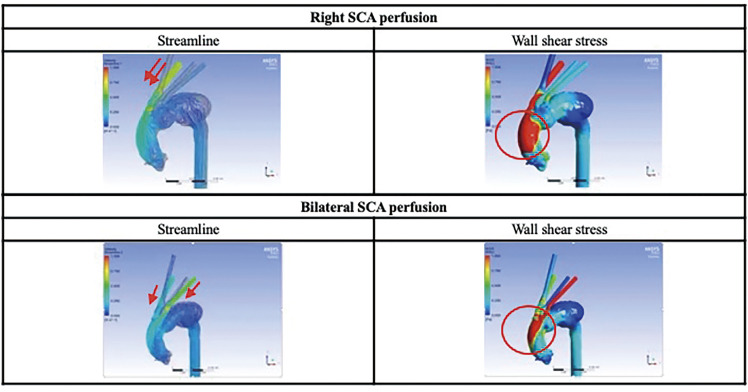
CFD analysis of SCA perfusion. Representative streamline patterns (left panels) and WSS distributions (right panels) in a representative patient during right SCA perfusion (upper panels) and a hypothetical bilateral SCA perfusion model (lower panels). Arrows indicate the direction of blood flow, and circles highlight regions of high WSS. High WSS regions were predominantly observed in the ascending aorta during right SCA perfusion and were reduced in the hypothetical bilateral SCA perfusion model, providing hypothesis-generating mechanistic insight into flow characteristics. CFD, computational fluid; SCA, subclavian artery dynamics; WSS, wall shear stress

In the bilateral SCA perfusion model, mixed flow patterns were observed, and the extent of high WSS in the ascending aorta was reduced (**Fig. 3**).

## Discussion

In this study, we demonstrated that quantitative assessment of aortic atheroma is significantly associated with postoperative CI after aortic arch surgery. In particular, the atheroma score of the ascending aorta showed strong predictive value and was identified as an independent risk factor. Importantly, this study demonstrates that quantitative atheroma assessment provides incremental value beyond conventional qualitative evaluation, which did not predict CI in our cohort. Furthermore, CFD analysis revealed that regions of high WSS were predominantly distributed in the ascending aorta, suggesting a potential mechanistic link between atheroma burden and embolic events.

Neurologic complications remain a major concern in aortic arch surgery despite advances in cerebral protection strategies.^2–4)^ Previous studies have identified atherosclerotic changes in the aorta as an important risk factor for embolic stroke.^5)^ However, preoperative assessment has largely relied on qualitative or subjective evaluation. In this context, the present study provides a reproducible quantitative method to assess atheroma burden using preoperative CT, enabling objective risk stratification.

Our findings suggest that the ascending aorta plays a critical role as a source of embolism during surgery. Most embolic events are thought to result from the dislodgement of atheromatous material due to aortic manipulation and high-velocity perfusion jets during CPB. The CFD analysis demonstrated that blood flow from the right SCA was directed toward the ascending aortic wall, where high WSS was observed, consistent with previous studies.^6,7,11)^ Elevated WSS has been associated with plaque destabilization and rupture.^8)^ These findings support the hypothesis that the combination of a high atheroma burden and increased hemodynamic stress contributes to postoperative CI. Notably, CFD analysis was used to provide mechanistic insight rather than direct risk prediction.

Interestingly, the bilateral SCA perfusion model reduced the extent of high WSS in the ascending aorta. This theoretical finding suggests that the perfusion strategy may influence WSS distribution; however, whether such modification reduces postoperative CI requires further clinical investigation. Previous clinical studies have also demonstrated the importance of cannulation strategies in reducing embolic complications.^12,13)^ Therefore, preoperative evaluation combining atheroma quantification and CFD-based analysis may provide a basis for future studies evaluating modified perfusion strategies.

From a clinical perspective, these findings suggest that patients with a high atheroma score in the ascending aorta may require particularly careful preoperative risk assessment and intraoperative planning. However, because the present study did not evaluate clinical outcomes after modifying cannulation or perfusion strategies, the clinical benefit of such strategy modification should be assessed in future studies.

### Limitations

The present study has several limitations. First, this was a retrospective, single-center study with a relatively small sample size. Second, only symptomatic CIs were evaluated, and subclinical events may have been underestimated. Third, the atheroma score represents the total plaque burden and is influenced by segment length; therefore, comparisons between segments should be interpreted with caution. In addition, the atheroma calculation assumes a circular aortic geometry, which may not fully reflect the actual vessel shape. The magnitude and direction of this measurement bias could not be quantified in the present study. More accurate plaque segmentation based on patient-specific vessel geometry may be required in future analyses. Furthermore, differentiation between atheroma and thrombus was not possible using the current method. We did not assess plaque composition, such as calcified or noncalcified plaques, using CT attenuation values. Future studies incorporating plaque morphology and Hounsfield unit-based assessment may improve embolic risk prediction. Finally, direct comparison with intraoperative epiaortic ultrasound was not performed.

## Conclusion

In conclusion, quantitative assessment of aortic atheroma, particularly in the ascending aorta, may help identify patients at increased risk of postoperative CI after aortic arch surgery. CFD findings were illustrative and hypothesis-generating and provided mechanistic insight into the relationship between perfusion flow and WSS distribution. Further studies are needed to determine whether modified perfusion strategies based on anatomical and hemodynamic assessment can reduce postoperative CI.

## Supplementary Materials

Supplementary Movie Blood flow streamline during right subclavian artery perfusion. The simulation demonstrates flow directed toward the ascending aorta, with subsequent complex flow patterns within the aortic arch.

Supplementary Table

## Declarations

### Data availability

The datasets generated and/or analyzed during the current study are available from the corresponding author upon reasonable request.
