# Supplementary material for: Preoperative Risk Stratification of Cerebral Infarction in Aortic Arch Surgery Using Quantitative Atheroma Assessment with Computational Fluid Dynamics
Source: Ann Thorac Cardiovasc Surg. 2026 Aug 14;32(1):26-00102. doi: 10.5761/atcs.oa.26-00102 (PMC13481124; doi:10.5761/atcs.oa.26-00102)
Supplement: Supplementary file 1 [file atcs-32-1-26-00102-s002.pdf]

Supplementary Table Length-normalized atheroma score

Variables	postoperative cerebral infarction group (n=8)	Non-postoperative cerebral infarction group (n=64)	P value	AUC
Zone 0 normalized score	56.8±42.5	26.9±41.2	<0.01	0.785
Zone 1 normalized score	177.6±199.6	41.2±76.0	0.051	0.717
Zone 2 normalized score	231.0±280.2	82.7±120.4	0.052	0.710
